# Diagnosis and management of non-CAH 46,XX disorders/differences in sex development

**DOI:** 10.3389/fendo.2024.1354759

**Published:** 2024-05-15

**Authors:** Zehra Yavas Abalı, Tulay Guran

**Affiliations:** Department of Pediatric Endocrinology and Diabetes, School of Medicine, Marmara University, Istanbul, Türkiye

**Keywords:** non-CAH 46, XX DSD, disorders/differences in sex development, primary glucocorticoid resistance, aromatase deficiency, testicular/ovotesticular disorders/differences in sex development, DSD, gonadal dysgenesis

## Abstract

Prenatal-onset androgen excess leads to abnormal sexual development in 46,XX individuals. This androgen excess can be caused endogenously by the adrenals or gonads or by exposure to exogenous androgens. The most common cause of 46,XX disorders/differences in sex development (DSD) is congenital adrenal hyperplasia (CAH) due to 21-hydroxylase deficiency, comprising >90% of 46,XX DSD cases. Deficiencies of 11β-hydroxylase, 3β-hydroxysteroid dehydrogenase, and P450-oxidoreductase (POR) are rare types of CAH, resulting in 46,XX DSD. In all CAH forms, patients have normal ovarian development. The molecular genetic causes of 46,XX DSD, besides CAH, are uncommon. These etiologies include primary glucocorticoid resistance (PGCR) and aromatase deficiency with normal ovarian development. Additionally, 46,XX gonads can differentiate into testes, causing 46,XX testicular (T) DSD or a coexistence of ovarian and testicular tissue, defined as 46,XX ovotesticular (OT)-DSD. PGCR is caused by inactivating variants in *NR3C1*, resulting in glucocorticoid insensitivity and the signs of mineralocorticoid and androgen excess. Pathogenic variants in the *CYP19A1* gene lead to aromatase deficiency, causing androgen excess. Many genes are involved in the mechanisms of gonadal development, and genes associated with 46,XX T/OT-DSD include translocations of the *SRY*; copy number variants in *NR2F2*, *NR0B1*, *SOX3*, *SOX9*, *SOX10*, and *FGF9*, and sequence variants in *NR5A1*, *NR2F2*, *RSPO1*, *SOX9*, *WNT2B*, *WNT4*, and *WT1*. Progress in cytogenetic and molecular genetic techniques has significantly improved our understanding of the etiology of non-CAH 46,XX DSD. Nonetheless, uncertainties about gonadal function and gender outcomes may make the management of these conditions challenging. This review explores the intricate landscape of diagnosing and managing these conditions, shedding light on the unique aspects that distinguish them from other types of DSD.

## Introduction

Disorders/differences in sex development (DSD) refer to conditions due to the discrepant development of chromosomal, gonadal, and phenotypic sex (1). Androgen overproduction caused by abnormalities in the adrenal cortex and gonads or exposure to androgens from an ectopic/exogenous source may affect the normal sexual development of the individual (2). Fetal exposure to androgens causes DSD in an individual with 46,XX chromosomes and may be diagnosed with ambiguous genitalia in the newborn (3).

Congenital adrenal hyperplasia (CAH) due to 21-hydroxylase deficiency is the most common etiology of 46,XX DSD, and other rare forms of CAH causing 46,XX DSD include 11β-hydroxylase, 3β-hydroxysteroid dehydrogenase, and P450-oxidoreductase (POR) deficiency (1).

Non-CAH 46,XX DSD are categorized as follows: (1) disorders with excessive amounts of endogenous androgens, such as primary glucocorticoid resistance (PGCR) and aromatase deficiency; (2) increased exogenous androgen exposure, such as gestational hyperandrogenism; (3) disorders/differences of gonadal differentiation [testicular (T)/ovotesticular (OT)-DSD, ovarian dysgenesis (OD)]; (4) others, classified as Mayer–Rokitansky–Küster–Hauser syndrome (MRKHS) (types I and II) and variants—complex syndromic disorders like cloacal exstrophy (4) (**Table 1**).

**Table 1:** Etiologies of 46,XX DSD.

Normal ovarian development			
Androgen excess			
Endogenous	Exogenous		
Congenital adrenal hyperplasia (CAH) • *CYP21A2, CYP11B1, HSD3B2, POR* gene mutations	• Exposure to synthetic androgenic progestins • Androgen-producing ovarian tumors (hilar cell tumors, arrhenoblastomas/androblastomas, lipoid cell tumors, and Krukenberg tumors)		
Aromatase deficiency • *CYP19A1* gene mutations			
Primary glucocorticoid resistance • *NR3C1* gene mutations			
Abnormal ovarian development			
Testicular DSD^a^	Testicular/ovotesticular DSD, gonadal dysgenesis		
• *SRY* translocation to X or autosomal chromosome	Increased expression of pro-testis genes	Decreased expression of anti-testis genes	Genes with unknown functions
• Gain of function in genes involved in the key testicular pathway^b^	*SOX9*, *SOX3*, and *SOX10*	*WNT4* and *RSPO1*	*NR5A1*, *NR2F2*, and *WT1*
Malformations causing 46,XX DSD			
Müllerian anomalies Mayer Rokitansky Küster Hauser syndrome (MRKHS) MRKHS variants: Isolated (type 1) • *WNT4* gene mutations^c^ (Müllerian duct failure and hyperandrogenism) and some candidate genes Syndromic (type II MRKHS) Vaginal atresia, cloacal anomaly, MURCS (Müllerian duct aplasia, unilateral renal agenesis, and cervicothoracic somite anomalies) • *GREB1L* gene mutations (uterovaginal agenesis, ovarian agenesis, and renal abnormalities) • *MKKS* gene mutations (McKusick–Kaufman syndrome: vaginal atresia/ stenosis, congenital heart disease, vesicovaginal fistula, and mesoaxial/ postaxial polydactyly)^d^ Complex syndromic disorders Cloacal exstrophy, Müllerian duct agenesis, vaginal atresia, and labial fusion			

DSD, disorders/differences in sex development.

a Approximately 80% of cases are caused by the translocation of SRY to the X chromosome, while 46,XX DSD in the remaining patients is caused by a gain of function in genes involved in the key testicular pathway.

b All known genetic causes of nonsyndromic 46,XX testicular DSD can also lead to 46,XX ovotesticular DSD.

c Monoallelic WNT4 gene mutations cause Müllerian duct failure and hyperandrogenism, while biallelic WNT4 gene mutations cause SERKAL syndrome (46,XX DSD with dysgenesis of kidneys, adrenals, and lungs).

d Bardet–Biedl syndrome 6.

**Figure 1:**
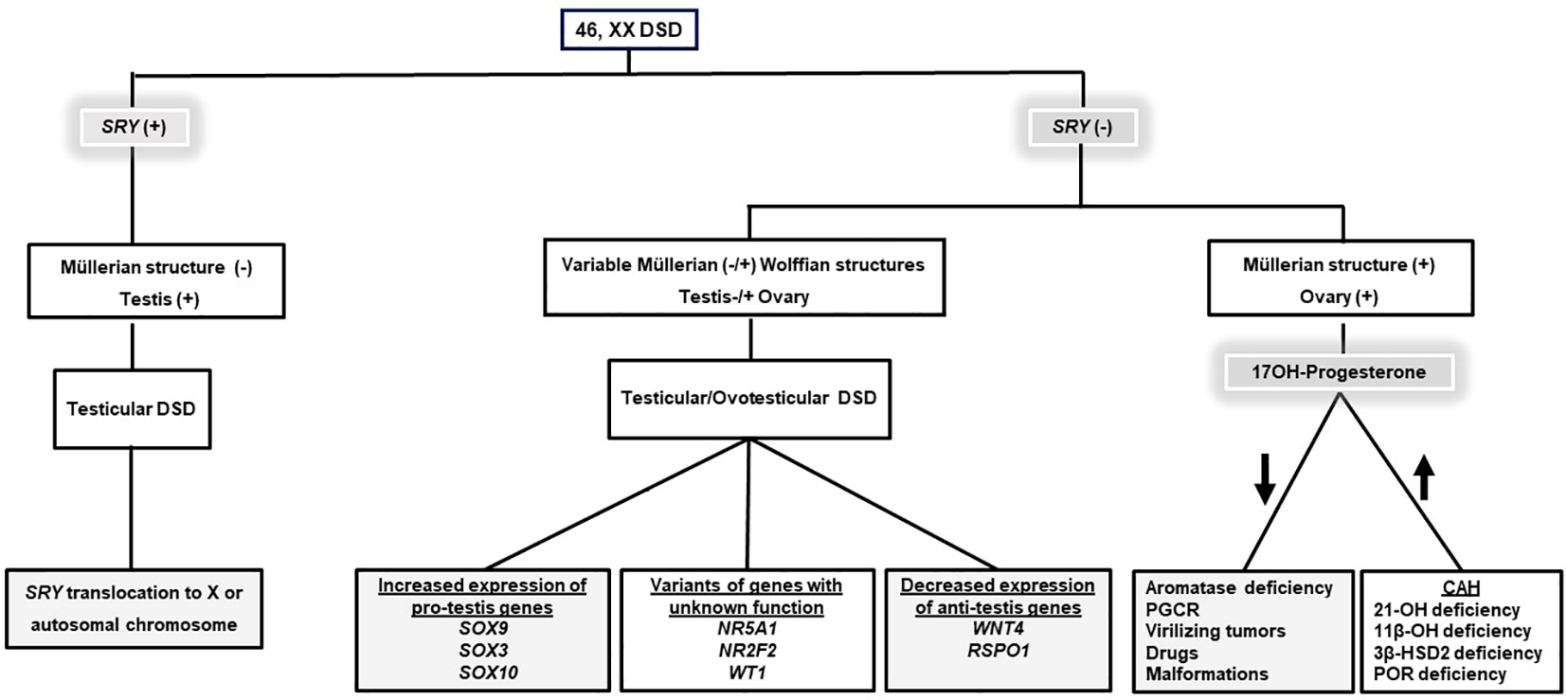
An algorithm for the differential diagnosis of 46, XX DSD. Gray boxes indicate the etiologies causing non-CAH 46, XX DSD. Information on SRY gene expression and 170H-Progesterone concentration is essential in the differential diagnosis of 46, XX DSD. DSD, Disorders/Differences in sex development; CAH, Congenital adrenal hyperplasia; 21-OH deficiency, 21α-Hydroxylase deficiency; 11β-OH deficiency, 11β-Hydroxylase deficiency; 3β-HSD2 deficiency, 3β-Hydroxysteroid dehydrogenase type 2 deficiency; POR deficiency P450 oxidoreductase deficiency, PGCR; Primary glucocorticoid resistance.

Differential diagnosis of these conditions and identification of the underlying etiology may affect the patient’s management and long-term prognosis (**Figure 1**).

In this chapter, we aim to emphasize the distinctive features of non-CAH 46,XX DSD and the challenges in the management of these conditions.

### Androgen excess in 46,XX with normal ovarian development

#### Primary glucocorticoid resistance

**Figure 2:**
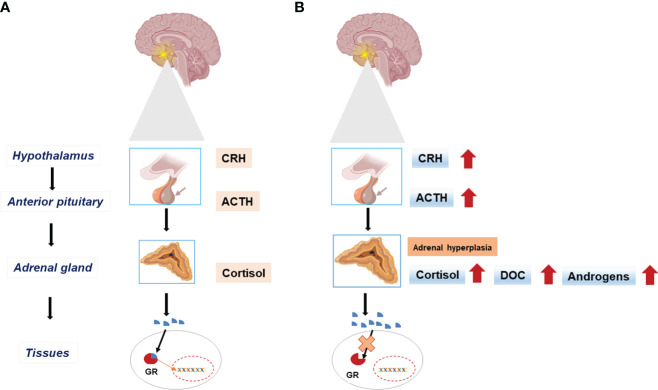
The hypothalamic-pituitary-adrenal (HPA) axis in physiologic state **(A)** and in Primary Glucocorticoid Resistance **(B)**. End-organ insensitivity to glucocorticoids (Cortisol) and impaired feedback mechanisms result in excess adrenocorticotrophic hormone (ACTH) secretion with increased circulating cortisol concentration. Excess ACTH leads to adrenal cortex hyperplasia and activates the synthesis of mineralocorticoids (DOC), and androgens. CRH, Corticotropin-releasing hormone; GR, Glucocorticoid receptor; DOC, Deoxycorticosterone.

Glucocorticoids (GCs) are synthesized in the zona fasciculata of the adrenal gland and function as the end products of the stress-responsive hypothalamic–pituitary–adrenal axis. They play an important role in both basal physiology and stress response (5). The effects of GCs are controlled by the glucocorticoid receptor (GR) (nuclear receptor subfamily 3, group C, member 1, *NR3C1*, MIM* 138040). GR is an intracellular receptor, a member of the steroid/sterol/thyroid/retinoid/orphan receptor superfamily, and it is widely distributed in various tissues (6). After binding to its ligand, this receptor communicates from the cytoplasm to the nucleus, and it controls the transcription rate of GC-responsive genes here (**Figure 2A**). Upon ligand-dependent activation of GR, these genes are influenced directly or indirectly (7).

GCs also have an important role in the treatment of inflammatory, autoimmune, lymphoproliferative, and allergic diseases. The pathologic or therapeutic effects of the GR, including genetic alterations in the human GR (hGR) gene, the development of GR ligands with selective GR actions, and disease-associated GR regulatory molecules, are extremely important (8).

Primary glucocorticoid resistance (PGCR) occurs due to inactivating mutations on the hGR gene *NR3C1* and causes systemic, partial GC insensitivity. Partial end-organ insensitivity to GCs and altered feedback mechanisms result in excess adrenocorticotrophic hormone (ACTH) production and thus increased cortisol concentrations (8, 9). Excess ACTH leads to adrenal cortex hypertrophy and increases mineralocorticoid (MC) and androgen synthesis (**Figure 2B**).

PGCR, also defined as Chrousos syndrome, is an extremely rare disease with a wide spectrum from asymptomatic to severe hyperandrogenism and MC excess (hypertension, hypokalemic alkalosis, and fatigue) (9). Acne, hirsutism, male pattern baldness, atypical genitalia, premature pubarche, precocious puberty, and subfertility may be observed due to hyperandrogenemia. PGCR may also cause irregular menstrual cycles and amenorrhea. The clinical findings of GC deficiency are subtle, like fatigue in childhood; however, growth retardation and hypoglycemia have also been reported (10, 11).

Plasma ACTH and serum cortisol concentrations are elevated in the majority of cases of PGCR. Hypertension and hypokalemia occur due to excess deoxycorticosterone (DOC) and increased cortisol availability at the MC receptor, causing a high ratio of cortisol concentration that overcomes the activity of 11β-hydroxysteroid dehydrogenase type 2 (HSD11B2). Most affected individuals present with hypertension, hypokalemia, and suppressed renin concentrations in childhood. The initial tests include the measurement of cortisol, androstenedione, testosterone, DHEAS, 11-deoxycortisol, and DOC concentrations (5, 12).

Although the circadian rhythm is maintained, it is primed to a high concentration in PGCR. The 24-h urinary cortisol (UFC) excretion is increased, and the serum concentrations of adrenal androgens (androstenedione, DHEA, and DHEA-S-) and MCs (DOC and corticosterone) are also increased due to compensatory ACTH elevation (8, 13).

A detailed medical history should be taken, and the signs of MC and/or androgen excess should be examined. Serum cortisol measurement and 24-h UFC excretion are required (two or three consecutive days). The HPA axis is resistant to dexamethasone suppression in PGCR; however, this may vary depending on the severity. Patients diagnosed with PGCR may respond to high doses of dexamethasone (14).

Differential diagnoses of PGCR include Cushing’s disease, pseudo-Cushing (depression and generalized anxiety disorder), conditions with increased cortisol-binding globulin (CBG) (pregnancy and estrogen treatment), hyperaldosteronism, essential hypertension, hyperandrogenism [polycystic ovary syndrome (PCOS), idiopathic hirsutism, and CAH] (12). Bone mineral density measurement is useful to differentiate PGCR from Cushing’s syndrome, and it is maintained in patients with GCR and increased in women due to androgen excess (15). In conditions with a clinical suspicion of PGCR, resistance can also be observed by the typical response of serum thyroid-stimulating hormone (TSH) to thyrotropin-releasing hormone (TRH) administration and/or the growth hormone response to insulin-induced hypoglycemia. These responses are compromised in Cushing’s disease (8).

The diagnosis of PGCR is confirmed by sequencing analysis of the *NR3C1* gene (8). To date, 56 variants of *NR3C1* have been described. Among these loss-of-function variants, 37 of them were missense, accompanied by frameshift and nonsense variants (16). Both biallelic and monoallelic variants that cause PGCR have been reported. A dominant negative effect on the wild-type GR is responsible for the effects of monoallelic variants causing GCR (8).

An estimate of the prevalence of *NR3C1* variants in a cohort of patients with adrenal hyperplasia, hypertension, and/or increased cortisol despite the absence of Cushing’s syndrome was first provided by Vitellius et al. (17). A total of 5% of these patients had monoallelic *NR3C1* variants. The variants [p.(R469*) and p.(R477S)] were identified in the DNA-binding domain, and the others [p.(R491*), p.(Q501H), and p.(L672P)] in the ligand-binding domain of hGR (17, 18). This prevalence rate indicates that many cases with *NR3C1* variants may be present but not diagnosed. Therefore, clinicians should advise *NR3C1* sequencing in patients with symptoms and signs indicative of PGCR (8).

A 3-year-old girl who was evaluated for hypertension, hypoglycemic seizures, and hypokalemia was reported by Tatsi et al. (19). The patient had findings of PGCR, and she was treated with antihypertensives, dexamethasone, and potassium. The heterozygous *NR3C1* variant p.(R714Q) was detected in this case, and it was first reported by Nader et al. (11). At the age of nearly 12 years, the patient was using high-dose dexamethasone, and she had radiological findings of hypertensive encephalopathy. In the exome sequencing (ES) analysis, Tatsi et al. identified two heterozygous variants: the one previously published by Nader et al. and a novel p.(E198*) variant. This was the first published case of biallelic *NR3C1* variants (compound heterozygous in the trans position) causing PGCR (11, 19).

In a Brazilian girl with clitoromegaly, urogenital sinus, and posterior labioscrotal fusion, a homozygous p.(V571A) variant was reported. This variant resulted in a marked reduction in receptor function. Although the phenotype might have been variable, severe GRα variants may manifest with mild 46,XX DSD (20). Next-generation sequencing (NGS) technologies have significantly contributed to the detection of pathogenic/likely pathogenic *NR3C1* variants. Considerable progress has been made in understanding the mechanism of a specific genetic defect in *NR3C1* that leads to a conformational change in hGRα (8). To date, no precise correlations between genotype and phenotype have been observed in PGCR (21).

Treatment of PGCR aims to reduce excess ACTH, suppress MCs, and/or regulate adrenal androgens. Supraphysiological doses of dexamethasone are used to elicit a physiological response in the presence of a poorly functioning receptor. Typically, the treatment starts with 0.25–0.5 mg/day and is adjusted gradually to suppress the ACTH, consequently decreasing androgens and controlling blood pressure. Depending on individual requirements, the dose of dexamethasone may be reduced. Hypertension may necessitate treatment with mineralocorticoid receptor antagonists (22). Thiazides and loop diuretics should be avoided due to the risk of hypokalemia. MC receptor antagonists can be used to manage hypertension, offering potential benefits owing to their anti-androgenic and potassium-sparing effects (8, 23).

#### Aromatase deficiency

**Figure 3:**
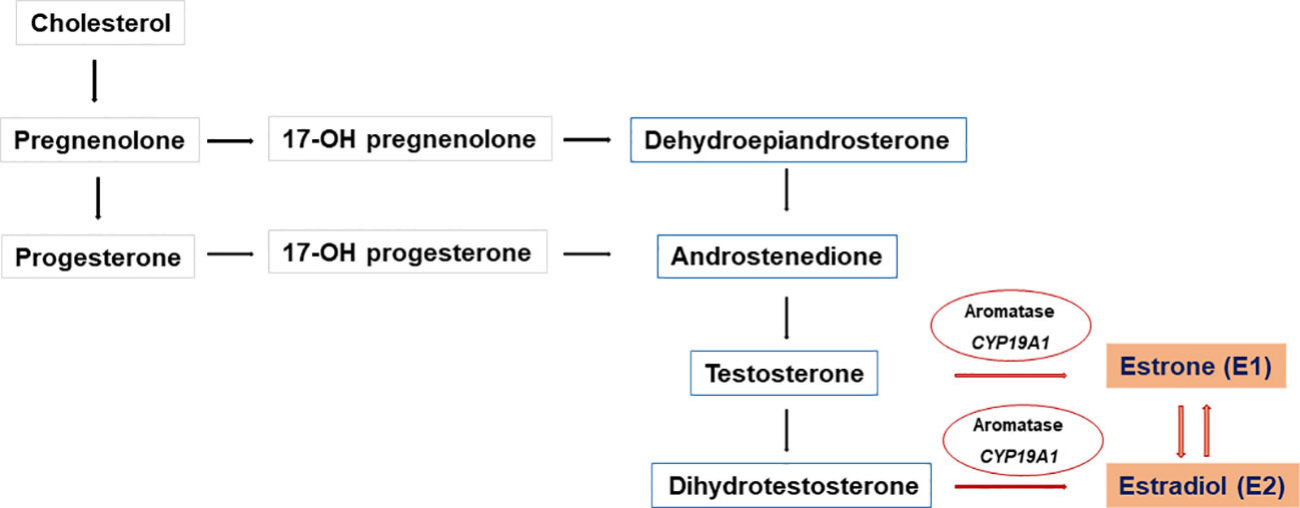
Biosynthesis of C18 steroids (estrogens). Aromatase (CYP19A1) converts C19 steroids (androgens) to C18 steroids (estrogens) Androstenedione, testosterone, and 16-α-hydroxy dehydroepiandrosterone sulfate are converted to estrone (E1), estradiol (E2), and estriol (E3), respectively E1 is converted to the biologically active E2 in target tissues by enzymatic processes with 17β-HSD activity.

Aromatase is a microsomal cytochrome P450 enzyme that catalyzes the conversion of C19 steroids (androgens) to C18 steroids (estrogens) (24). The androgenic precursors, androstenedione, testosterone, and 16-α-hydroxy dehydroepiandrosterone sulfate, were converted by aromatase to estrone (E1), estradiol (E2), and estriol (E3), respectively (25) (**Figure 3**).

Aromatase enzyme activity affects androgen metabolism and also influences the androgen–estrogen ratio in tissues (26). The aromatase enzyme is expressed in multiple tissues (ovary, placenta, adipose tissue, breast, brain, and bone). It is controlled by several tissue-specific promoters and has a role in the peripheral aromatization of androstenedione in adipocytes and skin fibroblasts (27). E1 has a weak estrogenic effect, and it is converted to estrone sulfate, which serves as a reserve for E1 in other tissues. E1 is converted to the active E2 in target tissues by enzymatic processes with reductive 17β-HSD activity. Androstenedione is the major substrate for aromatase activity in the target tissues. Aromatase has an important role in local estrogen production and estrogen synthesis from the ovary at the time of puberty (28).

Aromatase deficiency is caused by biallelic pathogenic/likely pathogenic variants in the *CYP19A1* gene (MIM*107910). Loss of function of this gene results in excess testosterone production by an otherwise normal ovary. *CYP19A1* is localized on chromosome 15q21.2 and consists of 10 exons (exon 1 has a role in tissue-specific gene expression) (26).

Shozu et al. were the first to describe aromatase deficiency, and subsequent studies determined that when the *CYP19A1* gene was knocked out in female mice, a male body habitus, small/polycystic ovaries, a small uterus, and infertility were observed (29). To date, many cases with variable clinical presentations have been reported, and 95 variants have been reported in *CYP19A1* (33 missense/nonsense) (30, 31).

Placental aromatase expression protects the mother from the virilizing effects of fetal adrenal androgens in physiologic pregnancy conditions. Disorders including POR deficiency, maternal androgen-producing tumors, and aromatase deficiency may cause virilization in the fetus and mother (32). In cases of aromatase deficiency, the placenta is unable to synthesize estrogen and large amounts of testosterone and androstenedione are transferred into the fetal and maternal circulation, causing virilization of the 46,XX fetus, and mother. This is also described as *placental aromatase deficiency* (29). Maternal virilization, such as increased hair growth, acne, and voice changes, becomes apparent after the second trimester of pregnancy, and generally, these symptoms resolve after the birth of the baby (32). However, it is worth noting that maternal virilization does not consistently manifest in every patient with aromatase deficiency (33, 34).

46,XX cases with aromatase deficiency may exhibit clitoromegaly, posterior fusion, scrotalization of the labioscrotal folds, and, in some infants, a urogenital sinus (26). Affected female individuals (46,XX) have Müllerian structures. In infancy, the histology of the ovaries is normal; however, due to FSH stimulation in aromatase deficiency, multiple, enlarged follicular cysts may be observed. At puberty, affected 46,XX patients have hypergonadotropic hypogonadism, they fail to develop female secondary sex characteristics, and exhibit progressive virilization. Plasma androstenedione and testosterone concentrations are elevated, with low or not measurable E1 and E2 concentrations. The ovaries enlarge and develop multiple cysts at puberty. Hypergonadotropic hypogonadism and the large multicystic ovaries respond to estrogen therapy, but treatment with an anti-androgen is required in some cases (32, 35).

Hypoplastic ovaries may also be observed in patients with aromatase deficiency. Therefore, hypergonadotropic hypogonadism in aromatase deficiency may develop secondary to impaired estrogen biosynthesis or hypoplastic ovaries (36–38). The clinical and molecular characteristics of aromatase deficiency in 46,XX patients are summarized in **Table 2**.

**Table 2:** Clinical and molecular characteristics of aromatase deficiency in 46,XX patients.

Molecular etiology and mechanism	Biallelic *CYP19A1* gene mutations—loss of function
Phenotypic characteristics	Virilization at birth (clitoromegaly, posterior fusion, urogenital sinus, a single perineal orifice)
Puberty	Absent or delayed (no breast development, no growth spurt, normal pubic and axillary hair, primary amenorrhoea, further increase in clitoral size)
Ovaries	Multicystic, enlarged, or hypoplastic
Bone age	Delayed
Other features	History of maternal virilization during pregnancy Metabolic symptoms^a^ (hyperinsulinemia, abnormal plasma lipids)
Hormonal findings^b^	
FSH LH E2 Testosterone	High Normal/high Very low High
Maternal serum screening - triple test^c^	Abnormal

FSH, follicle-stimulating hormone; LH, luteinizing hormone; E2, estradiol.

a Because female patients usually receive estrogen supplementation from puberty, the frequency of metabolic symptoms is unknown.

b These hormonal findings may not be seen in prepubertal children.

c A triple screen in pregnancy includes alpha-fetoprotein (AFP), human chorionic gonadotropin (HCG), and unconjugated estriol (E3).

Estrogen is involved in the development of secondary sexual characteristics and the regulation of gonadotropin secretion in women and causes epiphysial closure, bone mass maintenance, regulation of lipoprotein synthesis, and carbohydrate metabolism (39, 40). Bone development, metabolism, and immune function are affected by aromatase deficiency, as described in the follow-up of a small number of 46,XX cases with aromatase deficiency and in studies of aromatase knockout mice. Dyslipidemia and hyperinsulinemia have also been reported, which may reflect estrogen insufficiency but may also reflect specific actions of aromatase itself (41). The demonstration of normal psychosexual development in adolescent or adult patients with aromatase deficiency suggests that estrogen does not have a critical role in the sex differentiation of the human brain. The long-term prognosis of female patients with aromatase deficiency is poorly understood, and it is still not clear whether these patients are fertile (32).

Functional studies for the aromatase activity have shown severe loss of enzyme activity in all cases, other than approximately 1% activity for the p.(R435C) variant detected in a compound heterozygote with the p.(C437Y) variant (42).

Cases of partial aromatase deficiency have also been described. A homozygous p.(R435C) variant has been described in a 46,XX girl who presented with atypical genitalia in the neonatal period but had breast development in adolescence. A single deletion of phenylalanine (Phe234del) causing partial loss of aromatase activity has been described in a virilized 46,XX female patient with Tanner stage 4 breast development at puberty (37).

Lin L et al. discovered that, in aromatase deficiency cases, even minimal aromatase activity can lead to breast development and estrogen production, particularly with elevated androgens. While alternative pathways for estrogen synthesis may exist, it is uncertain if they occur in humans. Individual variability in enzyme regulation and degradation of compounds could influence estrogen and androgen responses. Patients with complete aromatase deficiency have not shown estrogenization at puberty. Functional studies indicate a correlation between aromatase activity and the extent of estrogenization, with 0.7%–1.5% activity achieving Tanner breast stage 2 and 16%–19% allowing progression to breast stage 4 and full uterine growth (37).

### Increased exogenous androgen exposure

#### Gestational hyperandrogenism

In a 46,XX case with normal ovarian and adrenal function, virilization may occur due to exposure to maternal androgens or synthetic androgenic progestins. The aromatase enzyme produced by the placenta converts androgens to estrogens; hence, high concentrations of maternal androgens may exceed placental aromatase activity and cause fetal virilization. Maternal luteomas or theca lutein cysts may result in maternal virilization during pregnancy (43). Androgen-producing ovarian tumors including hilar cell tumors, arrhenoblastomas/androblastomas, lipoid cell tumors, and Krukenberg tumors, may also cause virilization. Although extremely rare, androgen-secreting tumors of the adrenals can also occur during pregnancy (44).

Progestogens (or progestins), which include both bioidentical progesterone from plants and synthetic progestogens, are drugs with progesterone-like effects. They are commonly prescribed to reproductive-aged women for contraception, preventing threatened miscarriage and preterm birth. Adverse effects on reproductive development, like virilization in female infants, have been reported in case studies after exposure to progestogens in the first trimester. Similar effects have been observed in animal studies following *in utero* exposure to certain synthetic progestogens. More research is required to better understand the potential association of prenatal exposure to progestogens and adverse pregnancy outcomes, congenital malformation incidence, and longer-term health outcomes in prenatally exposed offspring (45).

### Disorders/differences in gonadal differentiation (abnormal ovarian development)

#### 46,XX testicular (T) DSD and 46,XX ovotesticular (OT) DSD

**Figure 4:**
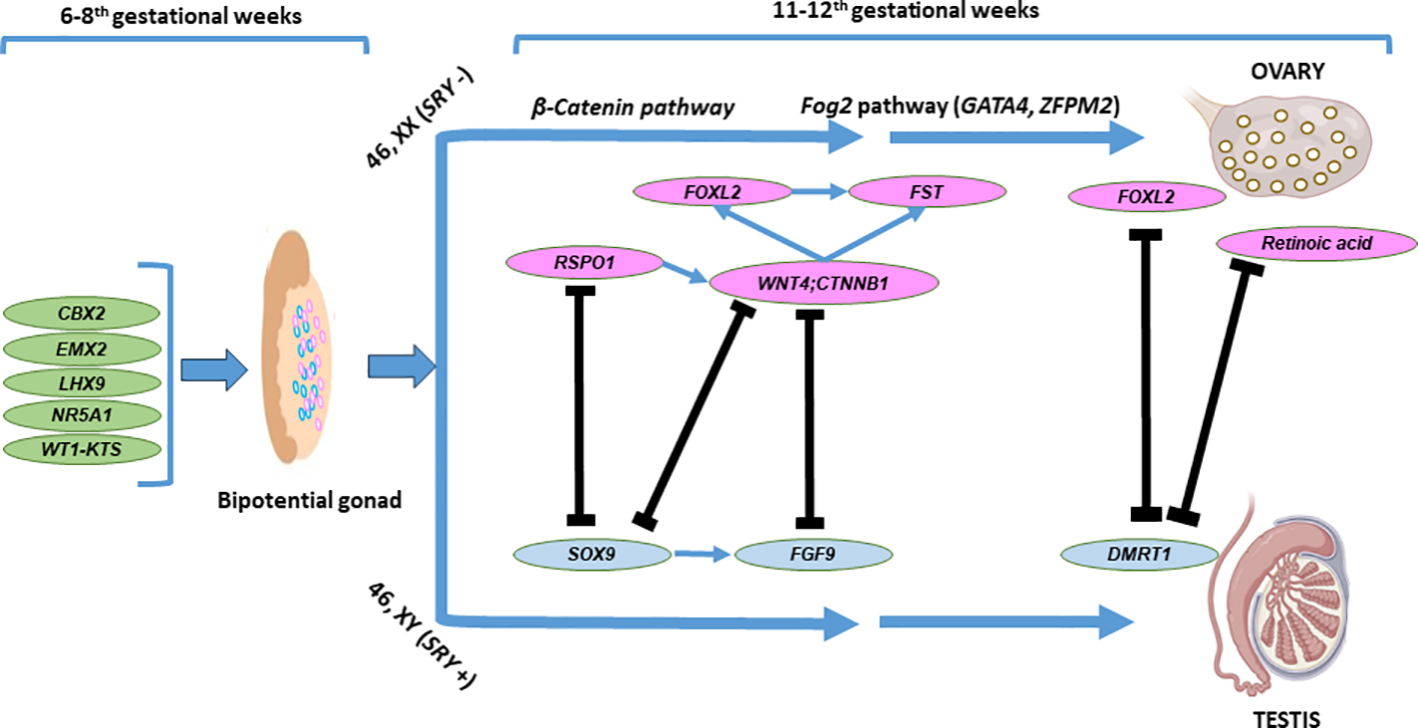
Basic genes and molecular pathways involved in ovarian development. The bipotential gonadal differentiates into ovary as a result of complex interactions between the testicular and ovarian developmental pathways Two components of the Wnt signalling pathway, WNT4 and RSP01 act synergistically to stabilise β-catenin encoded by *CTNNB1*, which promotes the expression of key ovarian genes, such as *WNT4* and *FST*. Fog2 pathway (*GATA4*, *ZFPM2*) is also involved in the ovarian development. The ovary specific genes *FOXL2*, *RSPO1*. *WNT4* and *CTNNB1* (β-catenin) counteract the testicular development through inhibition of *SOX9* and *FGF9* expression. *FOXL2* and ovarian retinoic acid is required in the adult ovary to suppress *DMRT1* expression and in the maintenance of granulosa and theca cell populations of the adult ovary.

The chromosomal sex determines the differentiation of the primitive gonad into an ovary or testis. However, this process relies on a complex network of genes in addition to the presence or absence of the *SRY* (sex-determining region on the Y chromosome), with either activation of the testicular pathway and repression of the ovarian pathway or *vice versa* (46). The fetal testis and ovary are indifferent (bipotential gonad) until the 6th gestational week (gw). Morphological changes that promote ovarian development occur at the same developmental time point when a 46,XY bipotential gonad commences the organization of the testis structure. Without *SRY*, ovarian differentiation begins on the 7th gw in female individuals. Despite the absence of visible morphological changes, gene expression patterns within the 46,XX somatic cells of the bipotential gonad have been described that drive the differentiation of granulosa cells and steroid-producing theca cells. The initiation signals for the differentiation of granulosa cells are not clearly understood. The specific genetic mechanisms controlling ovarian development are being clarified, and some of the regulators have been identified. The signaling factors *WNT4* and *RSPO1* increase and stabilize the expression of CTNNB1 (β-catenin). *CTNNB1* also maintains *WNT4* expression, represses male-specific *SOX9* expression, and promotes germ cell proliferation. In the 46,XX gonad, *WNT*, *RSPO1*, *CTNNB1*, *FOXL2*, and *FST* have a role in ovarian development and suppress testicular development. *RSPO1* enhances β-catenin signaling through *WNT4* in both humans and mice (47, 48). **Figure 4** illustrates ovarian development and maintenance.

In 46,XX T-DSD, gonadal development progresses in the direction of the testicular pathway, and the degree of testosterone and anti-Müllerian hormone (AMH) production determines the external and internal genitalia (49). The gonad may be either a normal testis or a dysgenetic testis. External genitalia may change from male to ambiguous genitalia. Azoospermia, absence of Müllerian structures, and absence of congenital anomalies are characteristic of the nonsyndromic form of 46,XX T-DSD. Approximately 15% of individuals with 46,XX T-DSD present with atypical genitalia at birth, while the remaining are present after puberty (2). Diagnosis is based on clinical, endocrinological, and genetic testing (cytogenetic and/or molecular genetic). Hypergonadotropic hypogonadism due to testicular failure is observed. The cytogenetic analysis reveals a 46,XX chromosome structure (50). Translocation of *SRY* to the X chromosome causes approximately 80% of XX T-DSD cases, particularly in patients with male external genitalia. 46,XX T-DSD in most other patients is caused by a gain of function in genes involved in the key testicular pathway. Molecular genetic diagnosis is rarely determined in 46,XX T-DSD, and OT-DSD with atypical genitalia (51).

In 46,XX OT-DSD, ovarian and testicular tissues are present, either as asymmetrically developed gonads or as ovotestes in one individual. Diagnosis is generally made by histology, but hormonal evaluation and imaging studies may be suggestive. Although the chromosomal structure is generally 46,XX, 46,XX/46,XY (chimerism) or 46,XX/47,XXY may be detected in rare cases. This is a phenotypic spectrum and is characterized by variable penetrance. Several families have been reported in which some 46,XX individuals have OT-DSD, some have T-DSD, and others are asymptomatic carriers (52). The estimated incidence of OT-DSD is <1/20,000 people and accounts for 3%–10% of all DSD phenotypes (53–56). 46,XX OT-DSD cases are often *SRY* negative, with few reported cases having a Yp;Xp translocation, including *SRY*. Although the molecular genetic etiology has not been established for the majority of cases, gain-of-function of pro-testicular genes and their regulatory regions or decreased expression of pro-ovarian genes have been detected in OT-DSD. Copy number variations (CNV) of *SOX* genes or regulatory regions of *SOX* (*SOX3*, *SOX9*, and *SOX10*) have also been described (53, 57).

An ovotestis is present in approximately two-thirds of the affected individuals with OT-DSD. The gonad may also appear as a streak gonad (non-functional dysgenetic tissue with fibrosis), and the characteristics of gonadal tissue may be detected on biopsy. However, the probability of bias in the sampling of a gonadal biopsy may miss the ovarian portion of the gonads (58). OT-DSD cases may have a uterus or hemiuterus; however, Müllerian structures are absent in 46,XX T-DSD. The development of reproductive organs is closely associated with the gonads, and due to the AMH effect, there is no fallopian tube or uterus on the side of the testes, whereas, on the side of the ovary, the fallopian tube, unicornuate uterus, and vagina may be seen (59).

The differences between 46,XX T-DSD, and OT-DSD are shown in **Table 3**.

**Table 3:** Differences between 46,XX T-DSD and OT-DSD.

	46,XX T-DSD	46,XX OT-DSD
Gonads	Testicular tissue	Both testicular and ovarian tissue present Any combination of the ovary, testes, or combined ovary and testes (ovotestes); streak gonads
Müllerian structures	Absent	Uterus or hemiuterus may be observed
Estradiol production	Absent	May produce estradiol
Fertility	Inability to generate sperm (Y chromosome must be intact for spermatogenesis)	Oocyte maturation may occur in some cases when intact ovarian tissue is present

T-DSD, testicular disorders/differences in sex development; OT-DSD, ovotesticular disorders/differences of sex development.

Recent evidence suggests that XX T-DSD and OT-DSD are the phenotypic spectrum of the same underlying defect in gonadal development (60, 61). All genetic etiologies causing 46,XX T-DSD can also lead to 46,XX OT-DSD (50, 53). *SRY* translocations, CNVs of *FGF9*, *NR0B1*, *NR2F2*, *SOX3*, *SOX9*, *SOX10*, and *SPRY2*, and sequence variants of *NR5A1*, *NR2F2*, *RSPO1*, *SOX9*, *WNT4*, *WNT2B*, and *WT1* are responsible for the genetic mechanisms associated with 46,XX T/OT-DSD (62).

## Molecular etiologies of 46,XX T-DSD and OT-DSD

### Presence of SRY

*SRY* (MIM*480000) encodes a transcription factor that is a member of the high mobility group (HMG)-box family of DNA-binding proteins. In the developing gonad, the presence of *SRY* promotes the activation of testicular pathways; hence, the translocation of *SRY* to the X chromosome or an autosome causes XX T-DSD. Approximately 20% of all 46,XX T/OT-DSD cases (~80% of T-DSD) are caused by the translocation of *SRY* to the tip of the X chromosome detected by FISH or chromosomal microarray (CMA). This is caused by an inappropriate recombination between the X and Y chromosomes during paternal meiosis (50, 61). *SRY*-positive XX T-DSD is generally caused by *de novo* translocation of the Y and the X chromosomes. In conditions with *SRY* translocation to the autosome or when fertility is preserved, sex-limited autosomal dominant inheritance is observed rarely (50, 54).

Y; autosome translocation is extremely rare. X;Y translocations leading to 46,XX T-DSD are frequently associated with nonallelic homologous recombination, whereas the underlying mechanism of Y; autosome translocations remains to be clarified. Unbalanced Y; autosome translocations can occur between two low-similarity sequences. Nonhomologous end joining may play a significant role in the development of Y-chromosomal translocations (63).

### *NR5A1* variants

*NR5A1* [(MIM*184757), steroidogenic factor 1 (*SF1*)] has a fundamental role in gonadal development and testicular differentiation, and it is an important etiology of 46,XY DSD. In addition to testis development, it also has a role in the activation of early ovary-determining genes, which occurs by upregulation of *NR0B1* (nuclear receptor subfamily 0 group B member 1, a repressor of *SOX9*) and β-catenin (**Figure 4**) (64). To date, 324 variants in *NR5A1* have been reported (31). In approximately 10%–20% of 46,XX T-DSD or OT-DSD cases, heterozygous variants in *NR5A1* have been detected (52, 64, 65). Almost all of these variants affect a single amino acid residue, suggesting gain-of-function variants that cause inappropriate activation of testicular pathways in a 46,XX gonad. A specific variant in the *NR5A1* gene [c.274C>T; p.(Arg92Trp); p.(R92W)] results in XX T/OT-DSD in some family members, while others are asymptomatic carriers (53). This variant represses the female-specific WNT signaling pathways (66). The exact mechanism is still not clear, but the hypothesis includes that p.(R92W) interferes with the NR5A1-mediated activation of ovarian development by an impaired interaction with β-catenin and by a loss of *NR0B1*-mediated suppression of *SOX9* (52). Different studies demonstrated that no loss of *SOX9* repression occurred and that *NR5A1* p.(R92W) and novel p.(A260V) variants did not decrease the *NR0B1* promoter activity (66). The variants disrupt the β-catenin-mediated activation of this promoter and result in increased repression of WNT signaling, resulting in reduced *NR0B1* activity. WNT signaling is controlled by the NR5A1/β-catenin complex in a dose-dependent manner. In addition, a p.(R92Q) variant in the *NR5A1* has been described in patients with and without OT-DSD (65). This variant did not show a reduced interaction with β-catenin (64). Pathogenic variants in *NR5A1* associated with 46,XX T-DSD, or OT-DSD are inherited as an autosomal dominant trait with variable expressivity and incomplete penetrance. A heterozygous parent (if fertile) will transmit the variant to 50% of the offspring—offspring who is 46,XX, and at risk for T/OT-DSD (50).

### Increased *SOX3* expression

*SOX3* (MIM* 313430, single exon) is localized in a highly conserved region of the X chromosome (Xq27.1) and is expressed in the brain, pituitary, and gonads, encoding a transcription factor very similar to *SRY* (44). Duplications or translocations in the regulatory regions of *SOX3* may cause an ectopic expression of *SOX3* in the 46,XX developing gonad, causing activation of testicular pathways (44). The first reports of 46,XX DSD due to *SOX3* duplications suggested that *SOX3* can act as *SRY* through increased expression. *SOX3* can act synergistically with SF1 to upregulate *SOX9* expression and activate testicular differentiation (67). Rare cases with *SOX3* duplication in 46,XX OT-DSD, or T-DSD have been reported (16).

46,XX OT-DSD has been reported in a patient with a 774-kilobase (kb) insertion that is translocated from chromosome 1 to a region 82 kb distal to *SOX3* (upregulation of *SOX3* expression) (68). In this case, the gonads were testis in ultrasound, but one of them was ovarian tissue on biopsy, causing an OT-DSD phenotype. The translocation, including *SOX3*, was inherited from a fertile mother (68). The different phenotypes of the mother and proband may be ascribed to differential X inactivation in the developing gonad. T-DSD has been suggested in the other five individuals with *SOX3*-associated 46,XX DSD, but histologically demonstrated in only one case (67).

To date, in all known individuals with CNVs in or around the *SOX3* gene with parental segregation analysis, it has been demonstrated that the disorder was caused by a *de novo* variant, and the risk of transmission to sibs is low (50). However, de Oliveira FM et al. described *de novo SOX3* duplication in two siblings with atypical genitalia, suggesting germline mosaicism as the etiology (69).

### *SOX9* duplication

The transcription factor encoded by *SOX9* (MIM*608160) functions downstream of *SRY* and is required for testicular development. Duplications of the *SOX9* locus and its upstream regulatory region have been reported in 46,XX T-DSD (57). DSD is caused by a gain-of-function CNV in the distal upstream regulatory region that duplicates one or more enhancer elements of the *SOX9* gene. Duplications can be localized up to 650 kb upstream of *SOX9* and induce testicular development by increasing the number of enhancers, which finally results in *SOX9* upregulation (70, 71). These duplications cause 46,XX DSD to varying degrees, and incomplete penetrance has also been documented (72). *SOX*9 duplications do not cause skeletal abnormalities. Hence, the gain- or loss-of-function variants in the *SOX9* gene appear to demonstrate a sex-limited manifestation/inheritance (71).

CMA detects large CNVs (including the regulatory regions around *SOX3* and *SOX9*) that cannot be detected by sequencing and small chromosomal rearrangements that may not be detected by karyotype. Small duplications or triplications in the regulatory regions of *SOX9* have been documented, which affect *SOX9* enhancers located up to two megabases (Mb) upstream of *SOX9*. A balanced translocation involving the 17q24.3 region has also been reported and should be observed in chromosome analysis (70). CNVs in or around *SOX9* have been inherited as autosomal dominant; however, only those individuals with a 46,XX chromosome are affected (50).

CNVs in the *SOX9* enhancer named RevSex cause XX sex reversal. The first case of 46,XX OT-DSD due to RevSex duplication demonstrated a male phenotype in affected XX family members. The father carrying the same variant was unaffected, which can be explained in the model where XY male individuals express high SOX9 levels during gonadogenesis, and further amplification of SOX9 expression does not deter from a pattern of typical male sexual development. Over the last decade, several cases of 46,XX OT-DSD caused by RevSex CNVs have been reported, exhibiting a range of genital presentations (typical to atypical genitalia). This phenomenon suggests that other genetic or non-genetic factors may contribute to the spatiotemporal expression of *SOX9* during development, leading to variable clinical outcomes (70, 73).

*SOX9* duplications more frequently result in 46,XX OT-DSD compared to 46,XX T-DSD. The two phenotypes have not been seen in the same family to date. To evaluate the risk of recurrence, the carrier status of the father should be analyzed. If parents are not carriers (*de novo*), the risk is no higher than the empirical risk in the general population. If the father is heterozygous for a CNV in or around *SOX9*, all reported 46,XY carriers have been fertile, anatomically male. One such 46,XY father (the father of a proband with OT-DSD) was reported to have inherited the duplication from his 46,XX mother. The risk of inheriting the CNV from the sibs is 50%. 46,XX individuals who carry the CNV are at risk of having 46,XX T, or OT-DSD. Individuals with 46,XY chromosomes will be fertile males (70).

### *WT1* variants

A DNA-binding protein containing four zinc fingers encoded by *WT1* (MIM* 607102) is essential for normal urogenital development (51). Pathogenic variants of *WT1* cause abnormal testis development, resulting in 46,XY DSD (74). The first report describing a *WT1* variant causing 46,XX T-DSD was published in 2017. In a male with microcephaly, dysgenetic testis, small uterus, and male external genitalia, the p.(Arg495Gly) variant was detected in *WT1* (75). Several cases of 46,XX T/OT DSD caused by pathogenic variants affecting the fourth zinc finger of *WT1* have been reported to date. This led to the identification of many male sex-determining genes and the suppression of *FOXL2* in a dominant-negative manner. Mutant *WT1* showed an interaction with β-catenin and upregulation of *SOX9* (75, 76). T-DSD is observed more frequently compared to OT-DSD in 46,XX cases, with frameshift and missense variants affecting the ZF4 domain of *WT1*. Of the nine individuals reported to date, four have histologically determined T-DSD, and two have OT-DSD. Of six reported individuals with *WT1*-related 46,XX T-DSD, only one had palpable gonads and typical male genitalia (75–77).

To date, all known individuals with a pathogenic *WT1* variant that causes 46,XX T-DSD whose parents have undergone molecular genetic testing have the disorder as a result of a *de novo* pathogenic variant, and the risk of transmission to the sibs is low (50).

### *NR2F2* variants

*NR2F2* (MIM*107773) is localized on chromosome 15q26.2, which encodes chicken ovalbumin upstream promoter transcription factor 2 (COUP-TF2). COUP-TFs are members of the steroid/thyroid hormone receptor superfamily. COUP-TF homologs, which have been cloned from humans, suggest that their protein sequences are highly homologous across species, defining functional conservation (62, 78). *NR2F2* is primarily expressed in mesenchymal cells (78). Compatible with the expression pattern of *NR2F2*, congenital heart defects are the well-known phenotypes associated with the variants of this gene (78). To date, 35 different variants have been described in *NR2F2*, and the majority of these are related to congenital heart defects (16).

In developing testicular tissue, *NR2F2* expression is observed in Leydig cells from 7 to 10 gw but is downregulated at 15 gw and repressed throughout fetal life. Previous studies concluded that *NR2F2* repression is important for fetal Leydig cell differentiation (62). According to rodent studies, the target genes of *NR2F2* in Leydig cells are *AMHR2*, *INSL3*, and genes encoding steroidogenic enzymes (62).

To date, four 46,XX T/OT-DSD cases with loss-of-function variants of *NR2F2* have been reported, suggesting that *NR2F2* is an anti-testicular gene. The first three cases with the frameshift variants were reported by Bashamboo et al., while one case with a 3-Mb deletion encompassing *NR2F2* was reported by Carvalheira et al. (79, 80). All four patients were 46,XX with testicular development. The mechanism underlying testicular development associated with *NR2F2* variants is not clear (62). Bashamboo et al. described that the two patients had the same 7-bp deletion, while one individual had a nearly identical 7-bp deletion. The variants were *de novo* in two patients, but the segregation of parents in the third case was not performed (80).

In a young man with XX OT-DSD, blepharophimosis–ptosis–epicantus syndrome (BPES), and coarctation of the aorta, a *de novo* 3-Mb deletion resulting in partial 15q monosomy of an evolutionarily conserved region was described. The deletion included the *NR2F2* and *SPATA8* genes, as well as three noncoding genes, three pseudogenes, and regulatory regions (79).

### Copy number variants around *NR0B1*

An 80-kb deletion involving the *NR0B1* and putative *MAGEB* regulatory regions in a patient with 46,XX OT-DSD was reported by Dangle et al. It has been hypothesized that the combination of a loss of one copy of anti-testicular *NR0B1* and overexpression of pro-testicular *MAGEB* resulted in testicular development in their patient (62, 81).

### Loss of function variants in genes repressing testicular pathways

The regulatory pathways controlling ovarian development have remained elusive in humans in contrast to testis formation. The pro-ovarian *WNT4/RSPO1* and beta-catenin pathways are activated in the absence of *SRY* (**Figure 4**) (82). Likely pathogenic/pathogenic variants in these genes may cause 46,XX T/OT-DSD and are often associated with abnormalities of other systems.

#### *WNT4* variants

*WNT4* (wingless-type MMTV integration site family member 4, MIM* 603490) controls the development of the female reproductive structure in the absence of *SRY* in 46,XX (83). *WNT4* has five coding exons and is localized on chromosome 1p36.12. The action of this gene occurs through the RSPO1-assisted canonical beta-catenin pathways. The mice study reported that excessive *Wnt4* supports feminization in male individuals, and insufficient *Wnt4* produces virilization in female individuals. WNT4 and its frizzled cell surface receptors have a role in the induction of female sex differentiation, and when both alleles of *WNT4* are inactive, SERKAL syndrome (sex reversal, kidneys, adrenal, and lung dysgenesis; MIM# 611812) occurs (84).

#### *RSPO1* variants

R-spondin proteins are agonists of the canonical WNT/β-catenin signaling pathway (85). Biallelic *RSPO1* (MIM*609595) variants cause a 46,XX T/OT DSD, palmoplantar keratoderma, and a predisposition to squamous cell carcinoma (86, 87). To date, nine variants in *RSPO1* have been reported, and only six of these are related to XX sex reversal (16).

## Management of 46,XX T-DSD and OT-DSD

The management of OT-DSD is complicated by numerous uncertainties about the etiology, gonadal function, and ultimate sex outcomes; therefore, a combination of medical, surgical, and psychological interventions must be implemented.

Inguinal or labioscrotal gonads, a hemiuterus, and a normal adrenal steroid profile in a virilized 46,XX infant should suggest OT-DSD. The AMH concentration, which reflects the amount of testicular tissue, is typically in the intermediate range in children with 46,XX OT-DSD. Since AMH tends to decrease in the initial weeks of postnatal life, serial AMH measurements after 1 to 2 months increase diagnostic certainty. AMH is invaluable for evaluating the presence of testicular tissue, its clinical utility decreases during the prepubertal period due to declining serum AMH with increasing intratesticular testosterone concentrations (53).

Normal serum gonadotropin concentrations in a XX OT-DSD suggest the existence of functional gonadal tissue. Unstimulated testosterone concentration is reliable during mini-puberty and from puberty onward. To adequately measure Leydig cell function in the first 2 to 3 weeks of life and childhood, an hCG test is required (88). However, a reliable functional test demonstrating the presence of ovarian tissue has not been described. In the study by Mendez et al., a human menopausal gonadotropin (hMG) stimulation test was performed on children with atypical genitalia. In patients with subsequent histologic OT-DSD, E2 levels increased above 80 pg/mL after the hMG challenge. All responders had ovarian or ovotestis, whereas none of the non-responders had histologic evidence of ovarian tissue. Nonetheless, further studies in larger numbers of patients are needed to confirm the sensitivity and specificity of this test (89).

Laparoscopic evaluation of genital structures and gonadal biopsies may be required for diagnosis. Nonetheless, in some situations, gonadal biopsies often do not reflect the characteristics of gonadal tissue and should not be regarded as a test for definitive diagnosis and decision making. Chromosomal analysis (exclusion of Y chromosome material by FISH or PCR) and NGS techniques (targeted gene panel, ES) focusing on the pathology of genes causing T/OT DSD and CMA for genomic imbalances are exclusively important for diagnosis (53).

Sex assignment is complicated in newborns with OT-DSD. Patient management must be highly individualized, and decisions should be made by a specialized multidisciplinary team and the parents. The severity of virilization, the approximate amount of gonadal tissue (testis or ovary), and the presence of the uterus may affect the final decisions. Gender outcome is unpredictable; hence, early genital surgery, like (partial) gonadectomy must be avoided in OT-DSD (53, 90). When gender identity is uncertain, GnRH analogs may be an alternative for use in childhood (91). The data about the fertility outcomes of OT-DSD are scarce. XX male individuals are infertile due to the absence of the Y chromosome. The characteristics of chimeric OT-DSD are unknown, but few reports indicate that XX males with testicular tissue had normal prepubertal progression, while testosterone concentrations decreased gradually. Hormone replacement may be required for these patients. In female individuals with ovarian tissue in place, menstruation may be observed, and some pregnancies have also been reported (92).

A gonadectomy may be required after puberty, provided that the gender identity is stable. In patients with a demarcated gonadal structure, gonad-sparing partial gonadectomy may be considered in the ovotestis (93). Monitoring of AMH and E2 concentrations is essential to ensuring that the relevant tissues have been completely removed. Nonetheless, it will be inevitable to completely extract the ovotestis.

Due to the possibility of persistence of functional gonadal tissue after partial gonadectomy in adolescents with OT-DSD, these patients should be followed up regularly. The residual tissue may cause clitoromegaly in female individuals (53).

The risk factors predisposing to malignancy are dysgenetic gonads, an intraabdominal gonad, and the presence of a Y chromosome. Knowledge about the risk of gonadal germ cell cancer (GCC) occurrence in OT-DSD is limited. Due to the absence of the Y chromosome, a low risk of GCC has been suggested, specifically in the gonadal tissues (94). The risk of GCC in OT-DSD is low compared to other DSDs. However, gonadoblastoma, seminoma, dysgerminoma, and yolk sac carcinomas have been described. This low-risk ratio likely reflects the fact that the majority of OT-DSD individuals are *SRY*-negative 46,XX cases (95). The ovarian reserve must be monitored in pubertal girls with OT-DSD to evaluate the possibility of oocyte cryopreservation (96).

Despite the identification of various genetic causes leading to defects in sex determination/differentiation, genetic factors alone cannot account for the diverse range of health or psychological issues that an individual with a DSD might encounter. Nonetheless, the detection of multiple developmental genes has increased our knowledge about the pathophysiology of DSD, which may help to follow the long-term effects of these etiologies and treatment outcomes in these individuals (58).

It is important to note that the management of these patients must be individualized, and decisions should be made with a team (endocrinologists, urologists, geneticists, and mental health professionals). Gonadal biopsy and early genital surgery, including gonadectomy, should be avoided in OT-DSD because of the unpredictability of rearing sex and adult outcome. The aim must be to provide comprehensive care that addresses both the physical and psychosocial aspects of the condition.

### 46,XX gonadal (ovarian) dysgenesis

46,XX gonadal (ovarian) dysgenesis (OD) is heterogeneous condition and may present with primary amenorrhea and infertility.

Multiple genes have been implicated in the etiology of OD, including *BMP15*, *PSMC3IP*, *MCMDC1*, *SOHLH1*, *NUP107*, *MRPS22*, *ESR2*, *SPIDR*, *FIGNL1*, and *ZSWIM7* (97, 98). Advancements in NGS techniques are progressively enhancing our understanding of 46,XX OD.

BPES is inherited in both dominant and recessive forms, and abnormal eyelids can be observed with OD (type I). Monoallelic variants of *FOXL2* (MIM* 605597) have been detected in 90% of BPES cases. Genomic rearrangements causing total or partial deletion of *FOXL2* account for approximately 12% of cases, while the remaining cases are caused by intragenic variants. Ovarian phenotypes are highly variable in female individuals with type I BPES, ranging from primary amenorrhea to irregular menstruation (99).

Perrault syndrome is a recessively inherited condition characterized by 46,XX OD, sensorineural hearing loss (in both sexes), and neurological findings in some cases. Mild intellectual disability with cerebellar and peripheral nervous system involvement may be observed. Perrault syndrome is clinically heterogeneous and is classified into type I (without neurological disease) and type II (progressive neurological disease) (100). Pathogenic/likely pathogenic variants in *HARS2*, *CLPP*, *LARS2*, *TWNK*, *ERAL1*, and *PROPR* may cause Perrault syndrome-related OD.

### Malformations causing 46,XX DSD

Isolated malformations of the internal genital tracts (vagina, uterus, and fallopian tubes) may be observed in some females, and these malformations may be related to the incomplete development of Müllerian structures or the presence of abnormal structures. A family history of malformations, which suggests a genetic etiology, may be detected in some patients, although the etiology is rarely elucidated. Aplasia or hypoplasia of the uterus and the fallopian tubes and a bicornuate/bipartite uterus that may be associated with malformations in other systems or tissues may occur. MRKHS (MIM%277000) is defined as the absence of a uterus and vagina in a phenotypically female 46,XX case. The two subtypes of MRKHS are an isolated type I and a type II with extragenital malformations. The majority of cases are sporadic. MURCS syndrome (MIM% 601076) includes Mullerian aplasia, renal aplasia, and cervical–thoracic somite abnormalities (101).

Molecular cytogenetic studies (CMA and MLPA) identified CNVs in different chromosomal regions, including the TAR susceptibility locus (1q21.1), chromosome 16p11.2, and 17q12 and 22q11.21 microduplication and deletion regions. The sequencing analysis of *LHX1*, *TBX6*, and *RBM8A* revealed other MRKH-associated genes. An analysis of *WNT9B* determined some causative variants in MRKHS (4).

In a group of patients with hyperandrogenemia, variants of *WNT4* are causative. Monoallelic *WNT4* variants cause Mullerian duct failure and hyperandrogenism, while biallelic *WNT4* variants cause SERKAL syndrome (46,XX DSD, dysgenetic kidneys, adrenals, and lungs) (102).

## Conclusion

The etiology of non-CAH 46,XX DSD includes a heterogeneous group of disorders. Progress in cytogenetic and molecular genetic techniques has significantly increased our knowledge about the etiology of these disorders. Nonetheless, uncertainties about the gonadal function and gender outcomes may still make the clinical management of these conditions complicated, and these patients must be monitored by an experienced multidisciplinary team.

## Author contributions

ZYA: Investigation, Writing – original draft, Writing – review & editing. TG: Investigation, Methodology, Project administration, Writing – original draft, Writing – review & editing.
